# Enhanced Cypermethrin Degradation Kinetics and Metabolic Pathway in *Bacillus thuringiensis* Strain SG4

**DOI:** 10.3390/microorganisms8020223

**Published:** 2020-02-07

**Authors:** Pankaj Bhatt, Yaohua Huang, Wenping Zhang, Anita Sharma, Shaohua Chen

**Affiliations:** 1 State Key Laboratory for Conservation and Utilization of Subtropical Agro-bioresources, Guangdong Province Key Laboratory of Microbial Signals and Disease Control, Integrative Microbiology Research Centre, South China Agricultural University, Guangzhou 510642, China; pankajbhatt.bhatt472@gmail.com (P.B.); 20183138021@stu.scau.edu.cn (Y.H.);; 2 Guangdong Laboratory of Lingnan Modern Agriculture, Guangzhou 510642, China; 3 Department of Microbiology, College of Basic Sciences and Humanities, G. B Pant University of Agriculture and Technology, Pantnagar 263145, India

**Keywords:** *Bacillus thuringiensis*, bioaugmentation, biodegradation, cypermethrin, kinetics, metabolic pathway

## Abstract

Cypermethrin is popularly used as an insecticide in households and agricultural fields, resulting in serious environmental contamination. Rapid and effective techniques that minimize or remove insecticidal residues from the environment are urgently required. However, the currently available cypermethrin-degrading bacterial strains are suboptimal. We aimed to characterize the kinetics and metabolic pathway of highly efficient cypermethrin-degrading *Bacillus thuringiensis* strain SG4. Strain SG4 effectively degraded cypermethrin under different conditions. The maximum degradation was observed at 32 °C, pH 7.0, and a shaking speed of 110 rpm, and about 80% of the initial dose of cypermethrin (50 mg·L^−1^) was degraded in minimal salt medium within 15 days. SG4 cells immobilized with sodium alginate provided a higher degradation rate (85.0%) and lower half-life (*t*_1/2_) of 5.3 days compared to the 52.9 days of the control. Bioaugmentation of cypermethrin-contaminated soil slurry with strain SG4 significantly enhanced its biodegradation (83.3%). Analysis of the degradation products led to identification of nine metabolites of cypermethrin, which revealed that cypermethrin could be degraded first by cleavage of its ester bond, followed by degradation of the benzene ring, and subsequent metabolism. A new degradation pathway for cypermethrin was proposed based on analysis of the metabolites. We investigated the active role of *B. thuringiensis* strain SG4 in cypermethrin degradation under various conditions that could be applied in large-scale pollutant treatment.

## 1. Introduction

Cypermethrin ((±)-α-cyano-3-phenoxybenzyl (±)-cis-trans3-(2,2-dichlorovinyl)-2,2-dimethylcyclopropanecarboxylate) is a synthetic pyrethroid that is commonly used against agricultural and household pests. Pyrethroids are applied worldwide to protect crop fields against insects and enhance agricultural production [1,2]. Pyrethroids also play a crucial role in the control of household insects, especially mosquitoes [3]. Low toxicity and adequate crop protection have motivated farmers to frequently apply synthetic pyrethroids [4]. Pesticides are important for pest control, but also affect human health and the environment [5,6,7]. Metabolites of synthetic pyrethroids have been reported in human urine samples, indicating their toxicity to living systems [8]. The microsomal enzyme carboxylesterase metabolizes these chemicals inside the human body [9]. 

Cypermethrin persistence in the environment varies according to the physiochemical conditions of the soil [10,11]. Microbial metabolism determines the fate of cypermethrin in soil and helps to reduce the concentration in the natural environment. Ester bond cleavage is the main route of cypermethrin degradation under laboratory conditions, via hydrolysis to produce alcohol and acid. The alcohol-containing intermediate is further converted into 3-phenobenzoic acid [12,13]. Cypermethrin is a non-polar compound with low water solubility and is therefore adsorbed onto the soil particles, whereas intermediate metabolites are mobile in soil. 3-Phenobenzoic acid is a toxic intermediate of cypermethrin and other pyrethroid degradations, which disrupts endocrine activity [14]. Several cypermethrin-degrading bacterial strains have been reported to effectively use the pesticide as a carbon and nitrogen source [15,16,17,18]. Microbial strains of genera *Bacillus*, *Pseudomonas*, *Acinetobacter*, *Roultella*, *Aspergillus*, *Candida*, *Trichoderma*, and *Cunninghamella* have been used for the degradation of cypermethrin and other pyrethroids [19,20,21,22,23,24]. These isolates can degrade up to 80% of cypermethrin in liquid media [21,22,23,24]. Mixed microbial cultures have been reported to degrade cypermethrin more rapidly than axenic culture [25]. However, little is known about the kinetic and metabolic fate of cypermethrin in soil. Soil bacteria exhibit different metabolic pathways for cypermethrin degradation, and this strategy can be effectively applied to remove environmental contaminants [26]. In this study, we investigated the role of *Bacillus thuringiensis* strain SG4 in cypermethrin biodegradation under conditions similar to the natural environment. 

## 2. Materials and Methods

### 2.1. Chemicals and Media

Technical-grade cypermethrin was obtained from the Department of Chemistry, G.B Pant University of Agriculture and Technology, Pantnagar, India. It was dissolved in acetonitrile to prepare a stock solution of 1 mg·mL^−1^, which was filter sterilized and kept in the refrigerator. Minimal salt medium (MSM; (NH_4_)_2_SO_4_ 2.0 g·L^−1^, Na_2_HPO_4_·12H_2_O 1.5 g·L^−1^, KH_2_PO_4_ 1.5 g·L^−1^, MgSO_4_·7H_2_O 0.2 g·L^−1^, CaCl_2_·2H_2_O 0.01 g·L^−1^, FeSO_4_·7H_2_O 0.001 g·L^−1^; pH 7.0) and nutrient broth (beef extract 1.0, yeast extract 2.0, peptone 5.0, and sodium chloride 5.0 g·L^−1^; pH 7.0) were used for the cultivation of pesticide-degrading soil bacterial strains. Analytical grade chemicals and solvents were used in this study.

### 2.2. Enrichment, Screening, and Identification of Cypermethrin-Degrading Bacteria

Cypermethrin-contaminated soil samples were collected from the agricultural fields in Pantnagar, Uttarakhand, India. Enrichment was carried out in MSM to isolate bacteria from collected soil samples. We used 1 g soil for bacterial isolation using the pour plate method. Bacterial colonies were transferred to 50 mL MSM containing cypermethrin (50 mg·L^−1^), and residue concentrations at various incubation intervals were quantified using high-performance liquid chromatography (HPLC) (Dionex Corp., Sunnyvale, CA, USA). The SG4 bacterial strain was isolated from the soil samples and selected for further studies based on its cypermethrin-degradation ability. The strain was identified based on a polyphasic approach using its morphological, biochemical, and molecular characteristics [12,27,28].

### 2.3. Effect of Temperature, pH, and Shaking Speed on the Degradation of Cypermethrin

To investigate the effects of temperature, pH, and shaking speed on the degradation of cypermethrin by strain SG4, a single-factor test was designed under different temperatures (28, 32, 36 °C), pH (5.0, 7.0, 9.0), and shaking speed (90, 110, 130 rpm) conditions. Strain SG4 was incubated in MSM (pH 7.0) containing 50 mg·L^−1^ cypermethrin at 32 °C at 110 rpm on a rotary shaker for 15 days. Each treatment was performed in triplicate with non-inoculated samples as the control. Residual pesticide concentration was determined by HPLC [13,29,30,31].

### 2.4. Effect of Immobilization on Cypermethrin Degradation

#### 2.4.1. Sodium Alginate Beads

Cypermethrin biodegradation was tested with immobilized bacterial culture in sodium alginate beads at 32 °C for 15 days. Sodium alginate beads were prepared by mixing 4% sodium alginate with homogenized bacterial culture(s). The mixture was poured drop by drop into pre-chilled autoclaved CaCl_2_ (0.4 M) through a needleless syringe to prepare fine beads (2 mm) inside the laminar airflow. Beads were stored overnight at 4 °C [32]. MSM (50 mL) was prepared in replicates in a 100 mL flask for biodegradation experiment and cypermethrin (50 mg·L^−1^) was added to the flasks along with 20 sodium alginate beads. We removed 1 mL aliquots of the broth from each flask on 5th, 10th, and 15th days and cypermethrin was extracted from the samples. Non-inoculated flasks spiked with cypermethrin served as the control and samples were quantified by HPLC. 

#### 2.4.2. Agar Discs

Agar discs were also used to analyze the cypermethrin biodegradation with immobilized bacterial isolates at 32 °C for 15 days. Agar solution (2.5%) was sterilized and cooled (44 °C) to prepare agar discs. Homogenized bacterial cultures were mixed with molten agar and poured into Petri dishes aseptically inside a laminar airflow [33,34]. Agar discs of equal size were prepared with a cork borer. We placed 50 mL MSM in a 100 mL flask and cypermethrin (50 mg·L^−1^) was added along with 20 agar discs. We removed a 1 mL aliquot of the broth from each flask on the 5th, 10th, and 15th days and cypermethrin was extracted. Non-inoculated flasks spiked with cypermethrin served as the control and all the extracted samples were quantified by HPLC.

### 2.5. FTIR Analysis of Cypermethrin Biodegradation

FTIR analysis was performed to estimate bond stretching during cypermethrin degradation (50 mg·L^−1^) in MSM (50 mL) inoculated with an active culture of strain SG4. Non-inoculated flasks with 50 mg·L^−1^ cypermethrin served as the control, and the experiment was repeated three times. For analysis, a 10 mL mixture of samples and acetone was prepared, and 10 mL ethyl acetate was added. After vortexing (1 min), the upper layer was collected in new tubes and sodium sulfate (5 g) was added. Samples were transferred to round-bottomed flasks and evaporated. Cypermethrin was collected in a dried round-bottomed flask with acetonitrile and analyzed using FTIR. Cypermethrin metabolites were extracted with acetonitrile after 15 days of inoculation. Bond stretching was analyzed in the F-IR system at a wavelength of 400–4000 cm^−1^, at the Department of Biophysics, G. B Pant University of Agriculture and Technology, Pantnagar, India [35,36]. 

### 2.6. Cypermethrin Degradation in Soil Slurry and Metabolite Identification

The soil slurry method was used to investigate cypermethrin degradation by strain SG4. We placed 50 g autoclaved soil in 250 mL flasks, and 20 mL minimal salt broth was added [37,38]. We then added 1 mL active bacterial inoculum into each flask containing 100 mg·L^−1^ cypermethrin and the experiment was performed in triplicate at 32 °C and 110 rpm on a rotary shaker. Cypermethrin residues were extracted at 0, 5, 10, and 15 days of incubation and flasks without the bacterial strain served as the control. Extracted cypermethrin was quantified through HPLC and metabolites were detected via gas chromatography–mass spectrometry (GC-MS; Shimadzu QP-2010 plus with Thermal Desorption System TD 20, New Delhi, India). 

### 2.7. Chemical Analysis

The cypermethrin residues of each experiment were analyzed in triplicate. Cypermethrin was extracted by adding 5 mL bacterial culture into 20 mL acetone in a flask. After shaking for 1 h, the mixture was carefully washed with 10 mL acetone and filtered through a Buchner funnel [39]. The filtrate was collected in a round-bottomed flask and pesticide residue was analyzed with HPLC attached to a C_18_ reverse-phase column and an ECD-3000RS electrochemical detector. A 10 μL mixture of acetonitrile and water (70:30, *v*/*v*) was used as the mobile phase at a flow rate of 1.0 mL·min^−1^.

### 2.8. Kinetic Analysis of Cypermethrin

The first-order kinetic equation was followed to fit experimental data of cypermethrin degradation kinetics as follows [40]:

(1) $$C_{t}=C_{0}e^{-kt}$$

(2) $$\ln C_{t}=C_{0}-kt$$

where *C*_0_ is the initial concentration of cypermethrin in the medium, *C*_t_ is the concentration of cypermethrin at time *t*, *k* is the degradation rate constant (day^−1^), and *t* is the reaction time. 

The biodegradation half-life (*t*_1/2_) of cypermethrin was calculated as

(3) $$t_{1/2}=\ln2/k$$

To calculate *k*, natural logarithm values of *C* were plotted against time *t*. Percent persistence values at different time intervals were calculated in comparison to cypermethrin recovered on the initial day (0) i.e., 1 h after the application (100%). The *k* of each concentration was computed from the slope of the line [41].

### 2.9. Statistical Analysis

Experiments were arranged as completely randomized designs with three replicates. Data were analyzed by one-way analysis of variance (ANOVA), and means were compared according to Bonferroni’s multiple comparisons test using IBM-SPSS software (version 22.0, IBM, New York, NY, USA). Statistical significance was determined by lowest significance differences (LSD) test at *p* < 0.05 to examine specific differences between treatments. 

## 3. Results

### 3.1. Enrichment of Bacterial Strain

Cypermethrin-degrading bacterial isolate SG4 was enriched and isolated from contaminated agricultural fields and characterized as Gram-positive rod-shaped bacilli. The morphological and biochemical characteristics of strain SG4 are provided in Supplementary Table S1. Strain SG4 efficiently degraded cypermethrin by using it as a source of carbon and nitrogen to grow. Phylogenetic analysis confirmed evolutionary similarities of strain SG4 with previously reported cypermethrin-degrading bacterial strains and was identified as *Bacillus thuringiensis* strain SG4 (accession number KT186610). 

### 3.2. Effect of Temperature, pH, and Shaking Speed on the Degradation of Cypermethrin by Strain SG4

**Figure 1:**
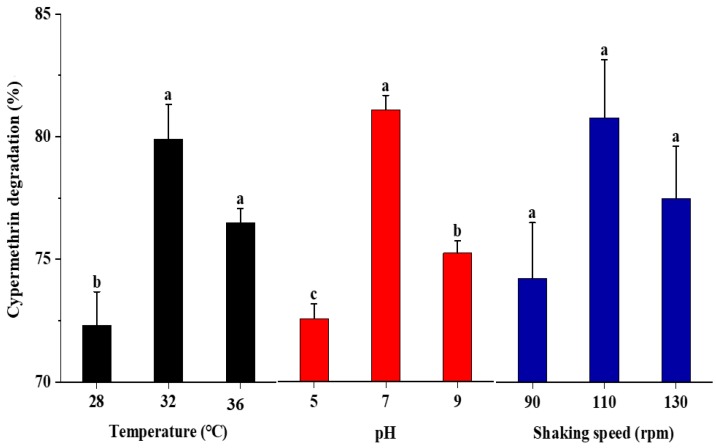
Effect of temperature, pH, and shaking speed on the degradation of cypermethrin by strain SG4. The data presented are mean ± standard errors of three independent experiments. Statistical analysis was performed by one way ANOVA of Duncan method, and different letters indicate significant differences (*p* < 0.05) between treatments.

The effects of temperature, pH, and shaking speed on the degradation of cypermethrin by strain SG4 were investigated in MSM (Figure 1). Strain SG4 effectively degraded cypermethrin under different conditions. The strain SG4 degraded 72.3%, 79.9%, and 76.5% of cypermethrin at 28, 32, and 36 °C, respectively, with 72.5%, 80.8%, and 75.1% degradation at pH 5.0, 7.0, and 9.0, respectively. For the shaking speed, 74.1%, 80.5%, and 77.3% degradation was achieved at 90, 110, and 130 rpm, respectively. The maximum degradation was observed at 32 °C, pH 7.0, and a shaking speed of 110 rpm. Under optimal conditions, strain SG4 degraded cypermethrin rapidly without a lag phase, with a *k* value of 0.1035 day^−1^ and a *t*_1/2_ of 6.7 days. In the non-inoculated control, the *k* and *t*_1/2_ values were 0.990 day^−1^ and 165.0 days, respectively (Table 1). The *t*_1/2_ value for cypermethrin significantly decreased when inoculated with strain SG4 in MSM. 

### 3.3. Cypermethrin Degradation with Immobilized Culture 

**Figure 2:**
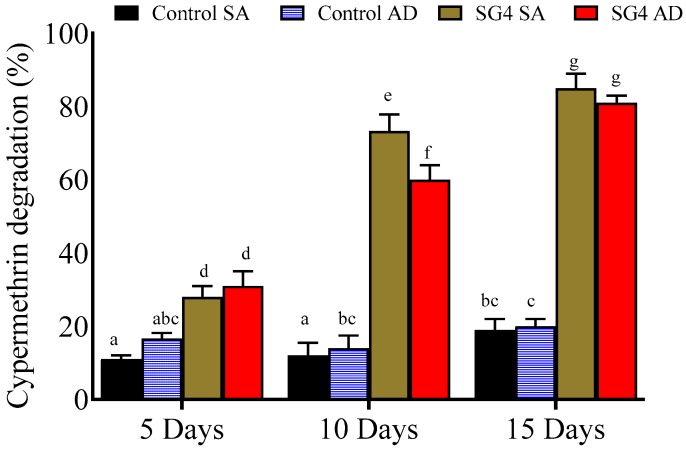
Effect of strain SG4 immobilization (sodium alginate and agar disc) on the degradation of cypermethrin. The data presented are mean ± standard errors of three independent experiments. Statistical analysis was performed by one way ANOVA of Duncan method, and different letters indicate significant differences (*p* < 0.05) between treatments. SA: sodium alginate, AD: agar discs.

The bioremediation capability of immobilized *B. thuringiensis* strain SG4 was determined in MSM with immobilized bacterial cells, using sodium alginate and agar disc as the matrix materials. Cypermethrin biodegradation by immobilized bacterial cultures in sodium alginate beads was found to be 28.0% and 73.0% on Days 5 and 10, respectively whereas maximum degradation of 85.3% was observed on Day 15 (Figure 2). The degradation with immobilized agar disc was 31.0%, 60.0%, and 81.0% on Days 5, 10, and 15, respectively. Cypermethrin degradation kinetics with immobilized strain SG4 demonstrated the better efficiency of these treatments (Table 2). Cypermethrin degradation with an external nitrogen source followed the first-order reaction model. Cypermethrin *t*_1/2_ in control with sodium alginate and strain SG4 treatments was 52.9 and 5.3 days, respectively. Agar-disc-based immobilized treatment resulted in cypermethrin *t*_1/2_ values of 50.9 and 6.4 days in control and strain SG4 treatment, respectively; the *k* values for sodium alginate control and strain SG4 were 0.0131 and 0.1330 day^−1^, respectively; whereas for the agar disc control and strain SG4 the *k* values were noted as 0.0136 and 0.1039 day^−1^, respectively. We confirmed that the degradation kinetics of sodium-alginate-based immobilized strain SG4 was more effective than the agar discs.

### 3.4. Degradation Kinetics of Cypermethrin in Soil Slurry

**Figure 3:**
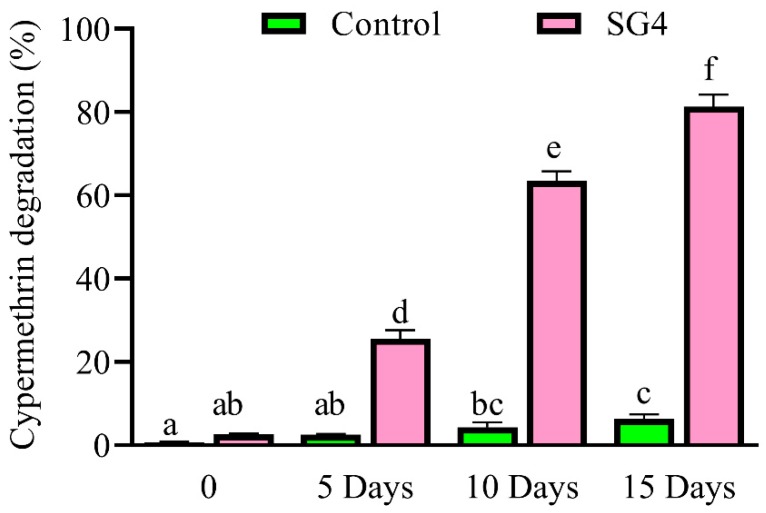
Cypermethrin degradation in soil slurry with strain SG4. The data presented are mean ± standard errors of three independent experiments. Statistical analysis was performed by one way ANOVA of Duncan method, and different letters indicate significant differences (*p* < 0.05) between treatments.

Cypermethrin degradation in soil slurry inoculated with *B. thuringiensis* strain SG4 followed first-order reaction kinetics. Inoculation of soil slurry resulted in 25.7% and 63.7% cypermethrin degradation on Days 5 and 10, respectively, which increased to 83.3% on Day 15 (Figure 3). The degradation kinetics of cypermethrin in soil slurry were reflected in the *t*_1/2_ value 177.7 and 0.7 days in control and strain-SG4-treated samples, respectively. The *k* values in soil slurry were 0.0039 and 0.113 day^−1^ in control and strain SG4 treatment, and the *R^2^* values were 0.999 and 0.968, respectively (Table 1). The observation of soil slurry suggested that *B. thuringiensis* strain SG4 has a strong cypermethrin degradation ability in agricultural fields.

### 3.5. FTIR Analysis of Cypermethrin Metabolites

**Figure 4:**
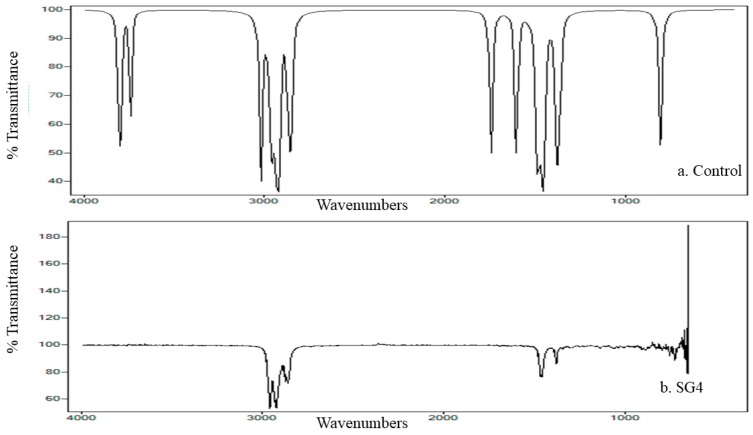
FTIR analysis of cypermethrin degradation with strain SG4 in minimal salt medium.

FTIR studies were performed to characterize bonding or stretching vibrations in cypermethrin under bacterial treatments. Different peaks in the control represented a variety of chemical bonds in the cypermethrin structure. Four peaks were observed in the control at 1000 to 2000 cm^−1^ and 3000 cm^−1^, which corresponded to stretching in the C–C, C–C, C–N, (C=O)−O−, and C–H bonds of cypermethrin. Conversely, peaks were absent in the strain-SG4-treated sample, revealing effective cypermethrin degradation (Figure 4). The results confirmed that cypermethrin degradation with strain SG4 is a systematic process that includes the opening and conversion of aromatic rings to give another intermediate compound.

### 3.6. Identification of Metabolites

**Figure 5:**
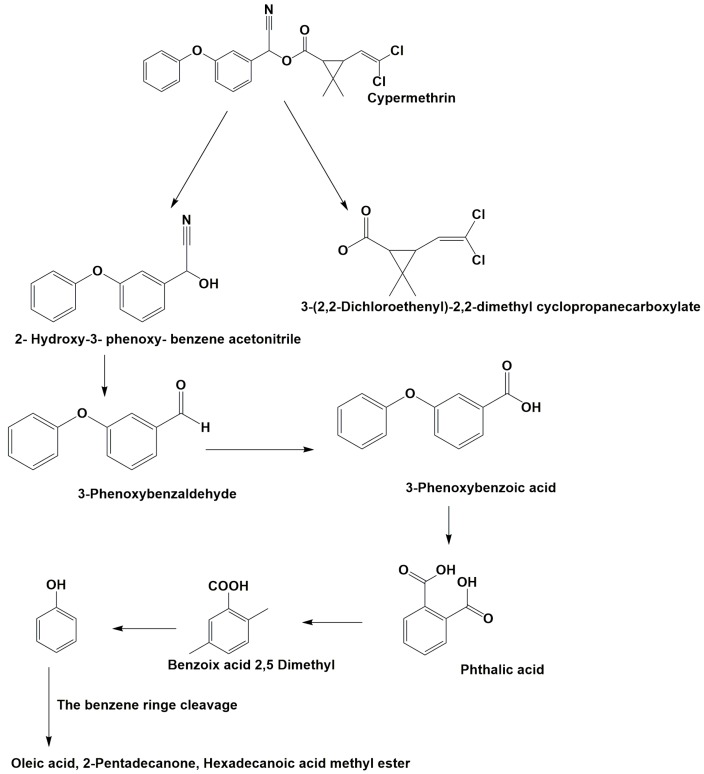
Metabolic pathway of cypermethrin degradation of strain SG4.

To identify cypermethrin degradation mechanism with *B. thuringiensis* strain SG4, HPLC and GC-MS were applied for metabolite characterization. HPLC-based quantification confirmed the degradation of cypermethrin on different days. The GC-MS chromatogram of cypermethrin with strain SG4 is shown in Supplementary Figure S1. Metabolites were characterized by comparison with the GC-MS compound library (Table 3). Bacterial strain SG4 degraded cypermethrin into smaller compounds with lower molecular weight. In soil slurry samples, the metabolite detected at retention time 4.123 min was identified as phenol, whereas the marker metabolite 3-phenoxybenzaldehyde was detected at a retention time of 13.728 with *m/z* 198. Table 3 describes the *m/z* values of the metabolites of strain SG4 in soil. The metabolic pathway was constructed based on the identified cypermethrin degradation metabolites (Figure 5). The metabolic pathway suggested that cypermethrin was initially degraded by the cleavage of the ester bond, followed by the cleavage of a benzene ring and subsequent metabolism. Analysis of metabolic products confirmed that no toxic intermediate was produced during strain-SG4-based degradation. Therefore, this strain can effectively and completely degrade cypermethrin via its metabolic pathway.

## 4. Discussion

Since the late 1980s, extensive cypermethrin applications in the agriculture and household sectors have led to toxic effects on organisms, necessitating its removal from contaminated environments. In this study, a cypermethrin-degrading bacterium was isolated from a pyrethroid-contaminated agricultural field. The bacterial isolate was characterized as *B. thuringiensis* strain SG4 (accession no. KT186610). Strain SG4 efficiently degraded cypermethrin in soil slurry and immobilized the culture with sodium alginate/agar discs. *B. thuringiensis* (Bt) is a bacterium well-known for its broad capabilities and it is widely used in biological control [42]. Bt has been reported for the degradation of cypermethrin and other pyrethroids [17,42,43]. However, the potential use of Bt in the bioremediation of pyrethroid-contaminated environments has not received adequate attention. Proteomics study of strain SG4 has confirmed differential protein expressions in the presence of cypermethrin [17]. Other cypermethrin-degrading microbial genera mainly include *Pseudomonas*, *Micrococcus*, *Acinetobacter*, *Aspergillus*, *Trichoderma*, and *Candida* [1,2,44,45,46]. These microbes use pyrethroids as the sole sources of carbon and nitrogen to fulfill their nutritional requirements [1]. Therefore, removal using a microbial system is more common compared with other modes of remediation. The key enzyme degrading cypermethrin and other pyrethroids belongs to the hydrolase family of enzymes known as esterases [47]. All the indigenous microbial strains with the potential for pyrethroid degradation are positive for esterase activity [48].

Temperature, pH, and shaking speed are considered important factors that influence the xenobiotic degradation ability of microbes [13,28,30]. Under the optimal conditions (32 °C, pH 7.0, and 110 rpm), strain SG4 degraded cypermethrin with a maximum degradation rate (*k* value of 0.1035 day^−1^). Therefore, these conditions were used for all the experiments. These results were consistent with findings reported by Chen et al. [13] who found that β-cypermethrin biodegradation likely occurs at neutral and alkaline conditions. Our results also showed that the maximum degradation of cypermethrin was achieved at pH 7.0. Strain SG4 effectively degraded cypermethrin at various conditions (28–36 °C and pH 5.0–9.0). This is a very important feature of an organism to be used for bioremediation in variable environments.

Cypermethrin and its intermediate metabolites were effectively detected and characterized by FTIR analysis [49]. The main cypermethrin peaks were found in the control, whereas these peaks were absent in strain-SG4-treated samples. The appearance of new peaks after bacterial degradation of cypermethrin showed that it was degraded by the cleavage of ester bond stretching. Microbial treatment of cypermethrin results in stretching of C=C chloroalkenes, benzene ring vibration, deformation in R–CH_2_–CN, and (C=O)−O bond [49]. Ether cyanate and ester bond stretching were also observed with *Bacillus* sp. SG2 after 15 days of cypermethrin degradation. Biodegraded cypermethrin with *Bacillus* sp. SG2 revealed major changes in the range of 100 to 1650 cm^−1^ and 2259 to 3431 cm^−1^ [12]. Photolytic degradation with similar peaks, also confirmed by FTIR, suggested that the basic mode of bond cleavage is the same for all processes [50]. Similarly, previous studies reported that cypermethrin is degraded through ester bond cleavage, releasing different intermediate compounds [15,21]. The FTIR analysis of pesticide residues can be confirmed on the basis of bond stretching with the corresponding peak area. The position of the bond cleavage site in pesticides can be confirmed using FTIR [51].

The study of an organism’s metabolic pathways is crucial for analyzing the biodegradation potential of a microorganism. Intermediate metabolites are sometimes more toxic than the parent compound [52,53,54]. We confirmed that strain SG4 produced non-toxic intermediates while effectively degrading cypermethrin. Our results aligned with previous reports on other bacterial strains [28,30,43]. Hydrolysis of cypermethrin was mediated via the cleavage of the ester bond, resulting in the formation of acid and alcohol [1]. The proposed degradation pathway of cypermethrin with strain SG4 is shown in Figure 5. The results revealed that cypermethrin was initially degraded into two metabolites: 3-(2,2-dichloroethenyl)-2,2-dimethyl cyclopropanecarboxylate and 2-hydroxy-2(3-phenoxyphenyl) acetonitrile. 2-Hydroxy-2(3-phenoxyphenyl) acetonitrile was then converted into 3-phenoxybenzaldehyde, which is considered a marker metabolic intermediate of cypermethrin and pyrethroid degradation. Previous studies have reported that this step is catalyzed by oxynitrilase [55,56]. The intermediate compound 3-phenoxybenzaldehyde appeared in small quantities during the initial phase of the experiment, but disappeared completely due to the effect of strain SG4. 3-Phenoxybenzaldehyde can be easily transformed via a bacterial system, as similarly reported by Birolli et al. [57]. 3-Phenoxybenzaldehyde can be converted into 3-phenoxybenzoic acid using the enzyme aldehyde dehydrogenase [58]. 3-Phenoxybenzoic acid can be converted into phthalic acid, which can further be converted into benzoic acid 2,5-dimethyl using the transferase family enzyme. This benzoic acid 2,5-dimethyl is further converted to phenol. These findings were similar to those previously reported in the literature [15]. The bacterium successfully grows by using and converting this toxic insecticide into non-toxic metabolic compounds. Similar results have been observed with different strains of bacteria and fungi [59]. Several degradation mechanisms for cypermethrin and other pyrethroids have been identified in bacterial strains of *Stenotrophomonas*, *Acinetobacter*, *Bacillus*, *Raoultella*, *Pseudomonas*, and *Brevibacterium*, and fungal strains *Aspergillus*, *Candidia*, and *Trichoderma* [2,15,19,20,59,60,61]. We propose the possible metabolic degradation pathway of cypermethrin with strain SG4 but, in natural conditions, the degradation could proceed via both biotic and abiotic reactions occurring in environmental conditions.

The addition of sodium alginate and agar discs with *B. thuringiensis* strain SG4 enhanced cypermethrin degradation compared with freely suspended cells. Tallur et al. suggested that agar disc and sodium alginate stabilize membrane permeability and offer protection against toxic intermediates [44]. During the study, we found the degradation of cypermethrin with sodium alginate (85.3%) to be higher compared to that with agar discs (81.0%). Increased surface area and stability associated with sodium alginate might have been the reason for this result. Immobilized microbial cells come into contact with microbes more frequently compared to freely suspended cells. An esterase-enzyme-based assay for pyrethroids suggested that the immobilization technique more completely degrades cypermethrin from the environment [32,62,63]. We also isolated efficient cypermethrin-degrading bacterial strain SG4 from contaminated agricultural fields, which then successfully degraded cypermethrin in soil slurry. Usually, isolated degrading microbes fail to degrade xenobiotics when used for the bioremediation of contaminated soil [28,64,65]. In this study, bioaugmentation of cypermethrin-contaminated soil with strain SG4 substantially enhanced its degradation and 83.3% of cypermethrin was removed from soil within 15 days. Kinetics analysis revealed that its *t*_1/2_ was reduced by 177 days in soil compared with soil without the SG4 strain. Unlike most bacterial isolates, strain SG4 has an exceptional ability to degrade pollutants in soil, highlighting its potential as a potent strain for the bioremediation of pyrethroid-contaminated environments.

## 5. Conclusions

*B. thuringiensis* SG4 uses cypermethrin as a carbon and nitrogen source, enabling its colonization of a niche area in a natural ecosystem where cypermethrin is present under limited nutrient conditions. Strain SG4 degraded cypermethrin in minimal medium, soil slurry, and in an immobilized culture assay with sodium alginate and agar disc beads. Our findings suggest that strain SG4 can efficiently degrade cypermethrin and similar ester-bond-containing pesticides. Our study of metabolites revealed that strain SG4 follows a complete degradation pathway to degrade cypermethrin. The degradation kinetics of cypermethrin under various treatments indicated that the strain could be used in large-scale applications in contaminated soil and water areas. Analysis with high-throughput techniques suggested that strain SG4 should be further explored to increase the understanding of the molecular machinery that regulates its degradation ability of toxic insecticides.

## Figures and Tables

**Table 1:** Degradation kinetics of cypermethrin with strain SG4 in MSM and soil slurry.

Treatment	Regression Equation	*k* (day^−1^)	*R* ^2^	*t*_1/2_ (days)
MSM	ln(C_t_/C_0_) = –0.0042x + 4.6074	0.0042	0.990	165.0
MSM + SG4	ln(C_t_/C_0_) = –0.1035x + 4.725	0.1035	0.954	6.7
Soil slurry	ln(C_t_/C_0_) = –0.0039x + 4.599	0.0039	0.999	177.7
Soil slurry + SG4	ln(C_t_/C_0_) = –0.1134x + 4.702	0.113	0.968	0.70

Initial cypermethrin concentration was 50 mg·L^−1^ in MSM and 100 mg·kg^−1^ in soil. The linear equation was derived from chemical data of *C*_0_ and *C*_t_ to calculate degradation rate constant (*k*), determination coefficient (*R*^2^) and half-life (*t*_1/2_) values. *t*_1/2_: cypermethrin disappearance time in days; *C*_0_ and *C*_t_: cypermethrin concentration at the initial and final stage of the experiment, respectively; MSM: minimal salt medium.

**Table 2:** Degradation kinetics of cypermethrin with immobilized culture.

Treatment	Regression Equation	*k* (day^−1^)	*R* ^2^	*t*_1/2_ (days)
MSM + SA	ln(C_t_/C_o_) = –0.0131x + 4.5868	0.0131	0.933	52.9
MSM + SA + SG4	ln(C_t_/C_o_) = –0.1336x + 4.7126	0.133	0.966	5.3
MSM + AD	ln(C_t_/C_o_) = –0.0136x + 4.5732	0.0136	0.859	50.9
MSM + AD + SG4	ln(C_t_/C_o_) = –0.1089x + 4.6867	0.1089	0.977	6.4

Initial cypermethrin concentration was 50 mg·L^−1^. A linear equation was derived from chemical data of *C*_0_ and *C*_t_, to calculate *k*, *R*^2^, and *t*_1/2_ values. SA: sodium alginate; AD: agar discs.

**Table 3:** Gas chromatography–mass spectrometry (GC-MS) analysis of the cypermethrin degradation metabolites.

Cypermethrin Degradation Metabolites Sequence	Retention Time (min)	Identified Metabolites	Molecular Weight (MW)	Chemical Structure
CP1	4.123	Phenol	94	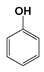
CP2	11.050	Benzoic acid, 2,5-dimethyl	150.17	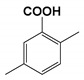
CP3	13.724	2-Hydroxy-3- phenoxy- benzeneacetonitrile	225	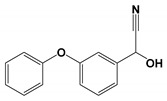
CP4	13.728	3-Phenoxybenzaldehyde	198	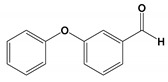
CP5	14.289	Phthalic acid	166.14	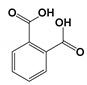
CP6	15.942	2-Pentadecanone	226.4	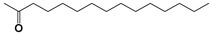
CP7	20.00	3-Phenoxybenzoate	228	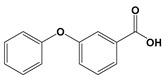
CP8	23.960	Cypermethrin	415	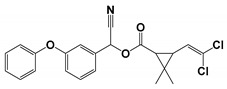
CP9	24.098	3-(2,2-Dichloroethenyl)-2,2-dimethyl cyclopropanecarboxylate	236	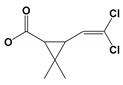

## Supplementary Materials

The following are available online at https://www.mdpi.com/2076-2607/8/2/223/s1.
