# Supplementary material for: Enhanced Cypermethrin Degradation Kinetics and Metabolic Pathway in Bacillus thuringiensis Strain SG4
Source: Microorganisms. 2020 Feb 7;8(2):223. doi: 10.3390/microorganisms8020223 (PMC7074683; doi:10.3390/microorganisms8020223)
Supplement: Supplementary file 1 [file microorganisms-08-00223-s001.pdf]

Supplementary data

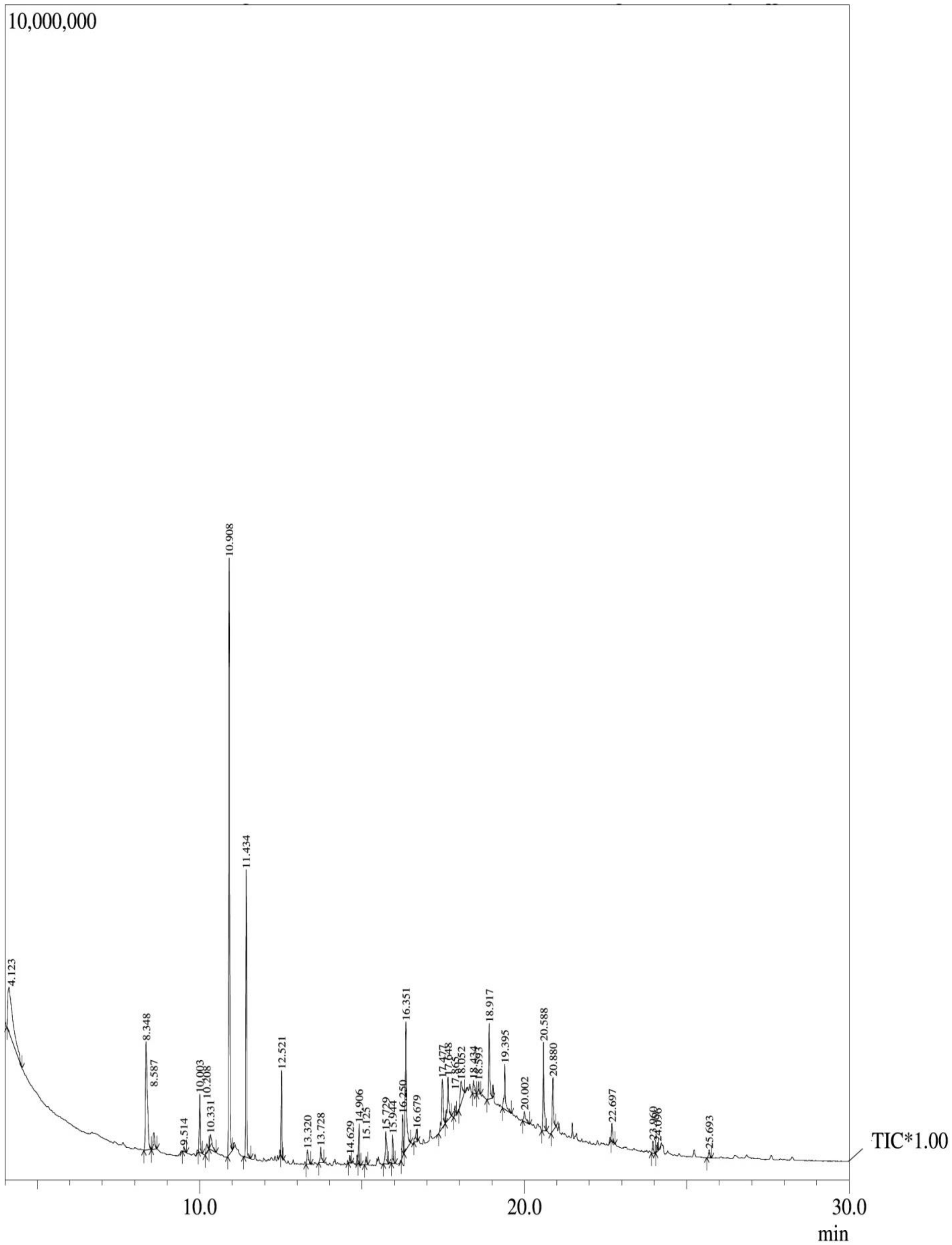

Figure S1. GC-MS chromatogram of cypermethrin with *Bacillus thuringiensis* strain SG4.

Table S1. Morphological and biochemical characteristics of *Bacillus thuringiensis* strain SG4.

Morphological and Biochemical Test	Response
Gram reaction	+
Catalase	+

Gelatin liquefaction	−
Lipase	−
Siderophore production	+
P-solubilization	+
IAA production	+
Cell shape	Rods
Arrangement	Scattered
